# Sleep Disorders in Huntington’s Disease

**DOI:** 10.3389/fpsyt.2019.00221

**Published:** 2019-04-12

**Authors:** Radoslawa Herzog–Krzywoszanska, Lukasz Krzywoszanski

**Affiliations:** ^1^General Psychology Unit, Chair of Psychology, Faculty of Pedagogy, Pedagogical University of Krakow, Krakow, Poland; ^2^Neurocognitive Psychology Unit, Chair of Psychology, Faculty of Pedagogy, Pedagogical University of Krakow, Krakow, Poland

**Keywords:** Huntington’s disease, neurodegenerative disease, sleep disorders, circadian rhythm disturbances, melatonin

## Abstract

Huntington’s chorea (Huntington’s disease, HD) is a genetic disorder caused by autosomal dominant mutation, leading to progressive neurodegenerative changes in the central nervous system. Involuntary movements such as chorea occur typically in HD patients, accompanied by progressive cognitive and psychiatric disturbances. Other common symptoms of HD are circadian and sleep abnormalities, which are observed from the earliest stages of the disease or even before the occurrence of clinical symptoms. The most common sleep problems reported by HD patients include insomnia, difficulties in falling asleep, frequent nocturnal awakenings, and excessive daytime sleepiness. Also, specific changes in sleep architecture have been identified in HD. In this paper, we review studies on sleep and circadian rhythm disorders in HD. We outline findings concerning sleep patterns and disturbances of circadian rhythms in HD patients, as well as the role of psychiatric disorders and motor disorders in HD patients’ sleep problems. We also discuss problems related to the different methods of diagnosing sleep disorders in HD. Furthermore, the adverse effects of medication used for the treatment of core HD symptoms as one of the sources of sleep disturbances in HD are emphasized. In conclusion, the diversity and complexity of the determinants of sleep and circadian rhythm disorders in HD are highlighted. Finally, the relevance of effective treatment to improve patients’ functioning and quality of life as well as the potential relief of their cognitive and emotional symptoms is addressed.

## Introduction

Huntington’s chorea (Huntington’s disease, HD) is a genetic disorder caused by autosomal dominant mutation, leading to progressive neurodegenerative changes in the central nervous system. The disease is caused by a dynamic mutation of the HTT gene, located on the short arm of the fourth chromosome. In the unaffected population, the number of CAG repeats in the HTT gene that encodes the huntingtin (HTT) protein varies from 6 to 35 (1). The mutant version of the gene contains from 36 to 250 repeats of this nucleotide sequence; as a result, a glutamine string present in the amino acid sequence of huntingtin encoded by the HTT gene is excessively elongated. A gradual accumulation of deposits of misfolded huntingtin and other proteins as well as the death of neurons in various areas of the brain are observed during the course of the disease. Neurodegenerative changes in HD primarily occur in the striatum and globus pallidus, but also in the cerebral cortex, cerebellum, amygdala, thalamus, and hypothalamus (2–8). The age of onset of HD symptoms strongly correlates with the number of CAG trinucleotide repeats in HTT. A higher number of CAG sequence repeats in the mutation carrier causes earlier onset of the disease, more severe cognitive impairments, severe progression of degenerative changes, and worse prognosis (9). The disease usually manifests in people aged 35–50, and it causes death within 15–20 years (10). It is estimated that HD frequency in Europe is 5–10 cases per 100,000 inhabitants (11).

Three main types of disorders described in HD are motor, cognitive, and psychiatric (12, 13). The most common motor symptoms in HD include chorea—uncontrolled movements of the head, neck, and limbs that may impede daily activities. Patients have difficulties maintaining balance and often stumble and fall. The clinical picture includes other motor symptoms such as bradykinesia, muscle rigidity, spasticity, myoclonus, dystonia, tics, ataxia, and athetotic movements (14).

The most common cognitive deficits in HD include attention (15, 16) and executive function disorders manifested by difficulties in taking decisions, planning and execution of complex activities, reduced flexibility of thinking and behavior, as well as a tendency to perseveration (17, 18). HD also involves implicit memory and learning disorders (19).

HD patients also develop psychiatric disorders that often occur before the onset of full-blown HD. Low mood and depression are revealed in a considerable number of the patients (20, 21), while anxiety disorders, dysphoria, emotional lability, apathy, and arousal are more rare (22, 23). In some patients, schizophrenia-like psychosis and paranoid symptoms have been observed (24).

The common symptoms of HD also include sleep disturbances (25). Up to 90% of patients report sleep problems that are evaluated as important by over half of them (26). Information about sleep disorders is also provided by spouses and caregivers. The number of CAG repeats in most studies did not correlate with the occurrence of sleep disorders (27); however, they were partially correlated with the duration of the disease and the severity of clinical symptoms (28). Studies have demonstrated that some sleep disorders are found in the very early phase of HD and even in carriers of the mutation at the premorbid stage (27, 29, 30). Many studies also show a high incidence of sleep disorders in other neurodegenerative diseases such as Alzheimer’s disease (AD), Parkinson’s disease (PD), or spinocerebellar ataxia (31–33).

Lack of sleep causes a decrease in mental alertness, irritability, attention disturbances, deceleration of reactions, and reduced ability for logical thinking. Sleep disorders considerably affect patients’ life activities, and their negative impact can be manifested especially in people with neurodegenerative disorders, who experience serious difficulties in everyday functioning due to the illness (29, 34, 35).

In this paper, we review the literature on sleep disorders and circadian rhythm disturbances in HD patients. We performed a search of online electronic databases (PubMed, MEDLINE, Scopus, and Google Scholar) that was most recently updated on October 10, 2018, followed by analysis of reference lists for additional articles.

## Sleep Pattern Disorders in HD

A typical sleep pattern in adults includes non-rapid eye movement sleep (NREM) divided into three stages (N1, N2, and N3): the sleep gradually deepens from N1 to N3, followed by rapid eye movement sleep (REM), during which suppression of postural muscle tone (atonia) is normally observed. NREM lasts about 60–90 min, REM lasts about 10–15 min, and the whole sleep cycle consisting of NREM and REM is repeated four or five times during a night’s sleep.

There is no homogenous pattern of sleep disorders in patients with HD (36) and problems can occur in various areas. The literature usually reports insomnia, increased sleep onset latency, decrease in total sleep time, frequent nocturnal awakenings (27, 28, 37–40), REM sleep disorders (27, 38, 41), increased motor activity during sleep (27), decreased sleep efficiency (37, 38, 41, 42), and excessive daytime sleepiness (43). The problems mainly concern impaired maintenance of sleep: patients frequently experience awakenings that are accompanied by electroencephalographic abnormalities (27, 38, 41). Some studies also show that awakenings may be accompanied by anxiety and choreiform movements (44). Reduced N3 stage and an increased sleep spindle density are observed in HD patients compared to healthy controls (28). It was revealed that HD patients spend less time in the deep sleep phase, as indicated by the higher percentage of the first stage of sleep compared to the control group (27). Compared to healthy subjects, individuals with HD also spend more time in the NREM stage than in the REM stage (37), which may be related to REM sleep disturbances in HD.

Reports presenting research concerning sleep disorders during the REM stage in HD patients are ambiguous. Some authors report a lack, shortening, or delay of the REM stage (27, 37, 38), but no REM sleep disorders were detected in other studies on HD patients (28).

In the study conducted by Arnulf et al. (27), reduced duration of the REM sleep stage was noted in mutation carriers without core symptoms, as well as in patients in the first stage of the disease, for whom it intensified with progression. These authors also claim that reduced REM sleep may precede chorea and provide an early marker of the disease. According to Arnulf et al. (27), reduced REM sleep atonia in HD patients with decreased duration of REM sleep and reduced eye movement density (38) indicates that REM sleep executive systems, which are mainly located in the brainstem, are damaged in HD. This postulate is consistent with findings showing atrophy and degenerative changes in the brainstem in HD (45, 46). Another cause of REM sleep disturbance may be the dependence of REM sleep mechanisms on cerebral blood flow and oxygen metabolism, both of which are impaired in HD (27).

HD patients also demonstrate behavioral disorders related to the REM sleep stage (RBD, REM sleep behavior disorder) (43). Skeletal muscle atonia is one of the characteristic features of REM sleep, and its absence can lead to sudden body movements, e.g., hitting, kicking, tossing, and turning during sleep; these phenomena are accompanied by realistic dreams, often provoking a sense of danger (47, 48).

Some authors (27) suggest that in HD a mutant form of huntingtin may accumulate in areas that control muscle atonia during REM sleep, thus leading to their failure. These areas include the dorsolateral pons, the locus coeruleus, the reticular formation of the medulla, the pedunculopontine tegmental nucleus, and the hypothalamus. Cell loss in the locus coeruleus (49) and in the hypothalamus (45, 50, 51) in HD has been confirmed in studies.

RBD carries a risk of self-injury or injury to other persons in the bed. Therefore, systematic interviews with HD patients and their caregivers about sudden movements and aggressive actions during their sleep may be useful in medical care. A significant role can be played by counseling on RBD, securing the sleep environment and pharmacological treatment.

## Circadian Rhythm Disturbances in HD

Changes in the sleep–wake cycle, such as difficulty in falling asleep and maintaining sleep in HD, are considered to be a manifestation of circadian rhythm sleep disorder (CRSD) (52). Some authors argue that the REM sleep delay in HD patients could also be a symptom of circadian dysfunction (26, 53). Moreover, circadian rhythm disorders may underlie excessive sleepiness in HD. Excessive daytime sleepiness (EDS) involves a strong subjective feeling of sleepiness, difficulty in maintaining wakefulness, and a tendency to fall asleep at the wrong time or in the wrong place. The most severe form of excessive sleepiness is sleep attacks, i.e., sudden, uncontrolled incidences of falling asleep, both while performing monotonous tasks and during intensified activity such as riding a bike: a short sleep lasting a few minutes is followed by sudden awakening with amnesia of the episode (54). The results of studies on the prevalence of EDS in HD patients vary depending on the applied research method (e.g., interview with the patient, the results of the Epworth Sleepiness Scale, or multiple sleep latency tests). In some research, daytime sleepiness in the HD group was similar to that in the control group (55), whereas in the study by Videnovic et al. (43), the frequency of EDS occurrence was high and affected 50% of the patients. Higher EDS results were correlated with depression; therefore, it is believed that effective treatment of EDS in HD should take into account the coexisting depression (43). EDS may also be caused by sleep disordered breathing, but only two studies have shown abnormalities of the sleep respiration in few HD patients (56, 57), while no differences between the HD patients and control subjects with respect to sleep respiratory variables have been found in other studies (58, 59).

The circadian rhythm in mammals is regulated by the suprachiasmatic nucleus of the hypothalamus (SCN), which controls melatonin synthesis in the pineal gland. Some researchers (60, 61) have described changes in circadian melatonin secretion in HD, both in the early and late stages of the disease. In another study, mean daytime melatonin levels did not differ between the patients with HD and controls, but the evening rise in melatonin level in HD patients was delayed compared to individuals without neurological disorders (61, 62). A more recent study (63) found reduced 24-h averaged plasma melatonin concentration, flattening of the circadian rhythm of melatonin secretion, and greater spread of melatonin onset time in premanifest HD and moderate HD patients compared with control subjects.

Circadian abnormalities were manifested in an HD animal model in transgenic R6/2 mice in which daytime activity was increased and nighttime activity was reduced (64, 65). Disturbed night–day activity in R6/2 mice worsened with the progression of the disease, finally leading to complete disruption of the behavior. Circadian sleep–wake cycle disturbances in R6/2 mice might be a result of the disruption of circadian rhythm regulation by SCN. SCN is a neural stimulator of the sleep–wake rhythm, and its activity is based on the cyclic expression of genes crucial for the internal clock—the so-called clock genes. Abnormal expression of mammalian Period2 (mPer2) and brain and muscle aryl hydrocarbon receptor nuclear translocator-like protein-1 (BMAL1) clock genes in SCN, as well as in the striatum and motor cortex, was observed in R6/2 mice. The authors (64) claim that the data from the studies on R6/2 mice suggest that abnormalities in SCN activity leading to sleep disturbances can also be observed in HD patients. An effect on the circadian regulation mechanism of the pathological processes that occur in HD has not yet been recognized. It is known, however, that HD is associated with serious damage to the hypothalamus, where the SCN is located (50, 66). The normalization of the circadian rhythm slows the increase of cognitive impairments in animal models of HD and thus potentially may also have a significant effect on the inhibition of the rate of disease progression and improvement of life quality in patients with HD (67).

## Psychiatric Disorders and Sleep Disorders in HD

Difficulties with falling asleep, shortness of sleep, and frequent awakenings may be the consequences of psychiatric disorders such as depression, mania, and anxiety, all of which affect many patients with HD. Depression with suicidal thoughts and tendencies develops in about 30% of people with HD and is often manifested even before the occurrence of motor symptoms (20, 21). According to some studies, the risk of depression and the rates of attempted suicide are higher in the period prior to the clinical HD phase and at its beginning (68). It is assumed that in most cases, depression in HD is endogenous and can result from damage to the medial caudate nucleus, from which projections go to the prefrontal and orbitofrontal areas. Research shows that depression in HD patients is associated with a reduction in glucose metabolism in the prefrontal and orbitofrontal cortex (69). Research on HD animal models also indicates a potential role of hypothalamus dysfunction as the neurobiological basis of depression in HD (70).

However, in addition to biological factors, psychosocial factors also play an important role in the development of depression in HD, which is an incurable disease that leads to disability and a significant reduction in quality of patients’ life. Coping with the disease and the associated stress may significantly affect the incidence of depression and anxiety symptoms in patients with HD. Studies have shown that the genetic diagnosis itself is a strong stressor that can lead to suicidal attempts and psychiatric disorders requiring hospitalization, not only in people who are diagnosed as defective gene carriers, but also among those in whom the result was negative (71).

Much clinical evidence indicates a strong relationship between depression and sleep disorders among different groups of patients with extrapyramidal system diseases, e.g., PD (72). Videnovic et al.’s (43) study shows more frequent sleep disturbances in HD patients with coexisting depression than in HD patients without depression symptoms.

In the study of Aziz et al. (73), sleep disorders reported by HD patients were associated with depression but did not correlate with the severity of motor, behavioral, and cognitive symptoms that are typical of HD. In contrast, a delayed sleep phase was associated with both depression and the deterioration of cognitive and everyday functioning. In another study (74), subjective reports of sleep problems were associated with a greater severity of depressive symptoms but were not associated with neurocognitive symptoms in premanifest and symptomatic HD individuals.

Some sleep disorder symptoms noted in HD patients, such as sleep onset latency, shortness of sleep, and frequent awakenings, are consistent with the symptoms of sleep disorders observed in depression. However, there are also significant differences between the pattern of sleep disorders in HD and sleep disturbances in depression, especially regarding REM sleep. Depression is characterized by a decreased latency of REM sleep, increased REM sleep, and increased number and density of rapid eye movements (75), whereas increased REM sleep latency, reduced REM sleep, and a decreased density of rapid eye movement are observed in HD (27, 37, 38). The similarity of some symptoms and differences in other symptoms of sleep disorders in HD and in depression most likely result from heterogeneous causes of sleep disorders in HD. They can be both a consequence of damage to brain areas responsible for sleep processes, e.g., the brainstem in reference to REM sleep and endogenous depression, and the result of an emotional reaction to stressful situations.

The course and quality of sleep in HD patients can also be adversely affected by arousal resulting from mania or anxiety disorders (24, 76). As much as 10% of HD patients may experience mania or hypomania episodes, and over 50% experience anxiety (21). Excessive arousal may be the basis for a pattern of sleep disorders that occurs in some HD patients, manifested by difficulties in maintaining sleep, lack of compensatory sleep during the day, and falling asleep later compared to people not suffering from insomnia (27).

A significant relationship between sleep disorders and psychiatric disorders emphasizes the need for their early detection and treatment in HD. Effective treatment of psychiatric disorders should also lead to sleep normalization.

## Motor Disorders and Sleep Disorders in HD

One of the causes of sleep problems in HD patients may be the involuntary movements that are one of the core symptoms of the disease (77, 78). It was originally believed that chorea ceases during sleep, but some studies have disproved this theory (37, 79). Patients are often not aware of their nocturnal movements, and information about their movements during sleep is obtained from their spouses or caregivers (72). As the disease progresses, further extrapyramidal symptoms develop, including bradykinesia, rigidity, myoclonus, and dystonic movements (involuntary movements of the limbs or the entire body). Rigidity and bradykinesia make it difficult to change position during sleep, and painful cramps and dyskinesias are the cause of awakening (73).

Studies demonstrate increased motor activity during sleep (55, 80, 81), including periodic limb movements (37, 82) and behavioral disorders during REM sleep (27) in HD patients compared to subjects not suffering from HD. More movements during sleep occurred in HD patients compared to patients with PD (79). Some HD patients suffer from restless legs syndrome (RLS), in which, due to unpleasant sensations, the patients move their legs to relieve the symptoms, as well as the syndrome of periodic limb movements in sleep (PLMS), characterized by brief, repetitive, and stereotyped limb movement during sleep. Both of these syndromes are associated with difficulties in falling asleep and frequent awakenings, which usually lead to constant fatigue, irritability, deterioration of concentration, and sleepiness during day (83). Comella (83) suggests that there is a genetic association between RLS and HD, but some studies have not revealed restless legs syndrome among examined HD patients (27). Evers and Stögbauer (84) hypothesize that dopaminergic transmission in HD may be reduced in parts of the basal ganglia, which are of particular importance for RLS. According to them, another explanation for the relationship between HD and RLS may be reduced sensitivity of the dopamine D1 and D2 receptors in HD patients with RLS. On the basis of their results, Savva et al. (85) postulate that RLS should be regarded as one of the early symptoms of HD that results from the neurodegenerative changes that occur in the disease.

## Methods for Diagnosing Sleep Disorders in HD

The most commonly used methods used for screening and diagnosis of sleep disorders in HD are self-report tools designed for sleep assessment in the general population, such as the Pittsburgh Sleep Quality Index (PSQI), IRLS, The Berlin questionnaire, and the Epworth Sleepiness Scale (ESS) (86). However, methods for sleep assessment in the general population may not evaluate the characteristic symptoms of sleep disorders of people with HD due to the specificity of the disease. Despite the prevalence of sleep and circadian rhythm disorders in HD, the Unified Huntington Disease Rating Scale (UHDRS), which is designed to assess the functioning of HD patients, does not include questions about sleep or the circadian rhythm (87). Research results indicate that in motor disorders, including HD, non-motor symptoms such as sleep disorders are often not reported by the patient or family (82, 88), which may impede the detection and treatment of sleep disorders. For both refining diagnoses and expanding the scientific knowledge about sleep disorders in HD, it seems advisable to include sleep questions in existing assessment tools and creating new HD-specific sleep scales. The detailed self-report questionnaire for assessment of sleep problems specific for HD patients, designed similarly to the questionnaires for assessment of sleep problems in PD patients, was developed recently (29). This questionnaire contains 45 questions grouped into four subcategories: quality of sleep (e.g., difficulty falling asleep or maintaining sleep), motor activity (e.g., painful muscle cramps in the arms or legs causing waking at night), abnormal nocturnal behavior (e.g., acting out dreams, injury to self or others while dreaming), and other aspects of disturbed sleep (e.g., nocturia, numbness, paresthesia, sleep apnea, and daytime somnolence).

The use of objective methods that provide data based on physical phenomena such as polysomnography and actigraphy could be potentially beneficial in the diagnosis of sleep disorders in HD, but it is associated with a number of difficulties. Polysomnography is considered a gold-standard diagnostic tool, but the availability of this method is significantly limited by the fact that it is complex, time-consuming, and expensive. Actigraphy is designed to assess the state of wakefulness and sleep on the basis of patients’ physical activity, but in the case of diseases with uncontrolled movements like HD, the test results may not be reliable. Studies using activity monitors show that during sleep, more movements and increased activity occur in HD patients compared to controls, which may mean that they either wake up at nighttime or make involuntary movements during sleep (55, 80). Fish et al. (79) observed that involuntary movements during sleep have a different character from those performed during waking—they are shorter and more fragmented. In combination with patients’ declarations that they did not wake up or did so only occasionally (55), the results support the hypothesis that nocturnal HD activity is associated with involuntary movements during sleep rather than waking up. In addition, low concordance of actigraphy results with EEG records and sleep diaries was demonstrated in the sleep study in HD patients carried out by Townhill et al. (81).

Contradictory results indicating overestimation of total sleep time by activity monitors in seven HD patients was reported in the study of Maskevich et al. (89), but the subjects in the analyzed sample were in the presymptomatic and early phase of the disease and had very little chorea. Those discrepancies indicate the need for caution in the interpretation of results obtained in studies of sleep and circadian rhythms in HD patients using activity monitors. However, Adams et al. (90) suggest that further development and improvement of accelerator-based research methods will allow them to be used to accurately monitor and diagnose sleep disorders in HD.

Piano et al. (91) investigated the coherence of self-report and laboratory-based sleep assessment in 30 HD patients; the concordance of subjective sleep evaluation based on two self-report measures, the Pittsburgh Sleep Quality Index and the Huntington Disease Sleep Questionnaire, was modest (Cohen κ = 0.375). Moreover, the results in both self-report measures demonstrated poor concordance with the Sleep Efficiently Index determined in a full-night laboratory-based video-polysomnographic recording (Cohen κ = 0.062 and 0.143, respectively) (91).

Proper diagnosis of sleep disorders in HD may require skillful combination of results from many different methods and integration of data from different sources as well as careful consideration of the specificity of this disease. The currently available methods should be considered imperfect as they require further development and improvement. There is also a need to develop diagnostic standards and guidelines for diagnosis of sleep disorders in HD.

## Pharmacotherapy and Sleep Disorders in HD

No systematic research has been conducted regarding the treatment of sleep disorders in HD patients; thus, the evidence base for the pharmacological treatment of HD is insufficient (92). The treatment is further complicated by the fact that many medications administered to HD patients to alleviate motor and psychiatric symptoms may change sleep architecture. Appropriate selection of drugs is also important because alleviating motor symptoms and depression themselves can improve sleep quality in HD patients.

Medications used in HD that are commonly associated with sleep disturbances are tetrabenazine, clonazepam, diazepam, riluzole, quetiapine, dosulepin, olanzapine, and venlafaxine. Drugs that may increase patients’ activation include amantadine, sodium valproate, and L-DOPA (52, 93). Antidepressants (especially venlafaxine) should not be used in HD patients suffering from RBD because they can aggravate the symptoms. In HD patients with RBD, it is possible to administer clonazepam, but in some cases, it is not well tolerated by HD patients and can cause respiratory depression (27). All dopaminergic drugs, including L-DOPA, could have sedative effects that may lead to excessive sleepiness (94). This undesirable side effect can be mitigated by dose adjustment or changing the drugs administered to patients. If given at night, some nondopaminergic drugs with sedative side effects that are used in chorea treatment, such as olanzapine, may help HD patients with insomnia (95).

In general, drugs that can interfere with sleep should be used with caution. In cases in which there is no opportunity to change medication, stimulant drugs should be given early in the day and drugs with sedative side effects should be given in the evening.

Apart from preventing negative effects on sleep related to drugs used in HD patients, some pharmacological agents can be used in order to improve the quality of sleep. The application of short-acting benzodiazepines for insomnia in HD patients may provide improvement; however, these medications need to be used with caution because of their potential side effects. Mirtazapine, which is a noradrenergic and specific serotonergic antidepressant, is recommended for HD patients suffering simultaneously from depression and sleep disorders (96). If the HD patient has daytime sleepiness, treatment with stimulants such as modafinil can be beneficial; its effect has mainly been evaluated in PD patients, in whom it improved daytime sleepiness and cognitive functions, was well tolerated, and did not impair motor functions (53). One study evaluating the effects of modafinil on HD patients showed (97) that it did not improve cognitive function or mood, but increased alertness.

Melatonin may also be beneficial in treatment because it promotes sleep and normalizes the circadian rhythm. Research has shown its effectiveness in improving the quality of sleep in AD patients (98) and PD patients (99, 100). In HD, delayed melatonin secretion rhythm and decreasing melatonin levels may contribute to progressive neurodegeneration. Therefore, the use of melatonin or its receptors’ agonists (ramelteon, agomelatine) could not only improve sleep quality but also enhance neuroprotection in HD patients (50). Regulation of the sleep–wake rhythm slows the increase of cognitive impairments in animal models of HD and thus potentially may also have a significant effect on the inhibition of the rate of disease progression and improvement of life quality in patients with HD (67).

Sleep quality in HD patients can also be improved by changing sleep behaviors. It is important to determine bedtime and wake time, avoid naps during the day, do regular physical exercise, maintain a proper diet, and limit the intake of caffeine, tobacco, and alcohol. In HD patients, relaxation training methods and cognitive-behavioral therapy can also be applied.

## Conclusions

Different patterns of sleep disturbances are observed in HD patients: insomnia, difficulties in falling asleep, frequent nocturnal awakenings, and excessive daytime sleepiness are the most common sleep problems reported by patients with HD. In several HD studies, specific changes in sleep architecture and in circadian melatonin secretion were identified in laboratory testing.

Sleep disorders in HD have diverse and complex determinants, the most significant of which includes damage to brain areas that are responsible for the proper sleep pattern and circadian rhythm regulation. Sleep and circadian rhythm disorders in HD might also be associated with psychiatric disorders, especially depression, mania, and anxiety disorders. Another group of factors related to sleep disturbances in HD are involuntary movements and increased motor activity during sleep, and other motor disturbances. Moreover, the pattern of sleep disorders in HD is often altered by the many drugs used to alleviate the core symptoms of the disease, a substantial number of which may adversely affect sleep.

Therefore, studies on sleep disorders in HD are associated with numerous difficulties, as is also the case with other neurodegenerative diseases. However, identification of sleep disorders and more comprehensive recognition of their causes are necessary to ensure adequate care and treatment for patients. Disorders of sleep and the circadian rhythm in patients with HD can contribute to exacerbation of symptoms such as irritability and anxiety, as well as cognitive disorders that can interfere with daily functioning more than the motor symptoms. The effective treatment of sleep disorders in HD patients may play an important role in improving the quality of their life, helping them cope with everyday problems, and relieving the cognitive and emotional symptoms. It should also have a great impact on the quality of sleep of caregivers and thus create the opportunity to delay patients’ institutionalization. More detailed knowledge of sleep problems in HD can also provide more profound insight into the nature of the neurodegenerative processes that occur in the disease (52).

Various methods are used for diagnosis of sleep disorders in HD patients. The most frequently used are self-reporting tools designed to assess sleep in the general population. However, the results obtained with these methods show low consistency with the results of laboratory sleep assessment, which makes their use in HD patients limited. Moreover, the proper diagnosis of sleep disorders in HD may require consideration of the specificity of the symptoms of this disease. The use of objective methods such as polysomnography and actigraphy might have potential benefits in the diagnosis of sleep disorders in HD patients, but the availability of polysomnography is limited because it is complex, time-consuming, and expensive, and the results of actigraphy may not be reliable in the case of diseases with uncontrolled movements, such as HD. Integration of data from various sources and results obtained from many different methods should allow the most comprehensive diagnosis of sleep disorders and circadian rhythm disorders in HD patients. The need to develop diagnostic standards and guidelines for the diagnosis of sleep disorders in HD should be considered one of the important challenges in improving the care of HD patients.

## Author Contributions

RH conceived and designed the study. RH and LK performed literature search and selection. LK created a database of literature relevant to the study. RH and LK wrote sections of the manuscript. Both authors contributed to manuscript revision and read and approved the submitted version.

## Conflict of Interest Statement

The authors declare that the research was conducted in the absence of any commercial or financial relationships that could be construed as a potential conflict of interest.
